# Intranasal Delivery of Camptothecin-Loaded Tat-Modified Nanomicells for Treatment of Intracranial Brain Tumors

**DOI:** 10.3390/ph5101092

**Published:** 2012-10-15

**Authors:** Hiroyuki Taki, Takanori Kanazawa, Fuminari Akiyama, Yuuki Takashima, Hiroaki Okada

**Affiliations:** School of Pharmacy, Tokyo University of Pharmacy and Life Sciences, 1432-1 Horinouchi, Hachioji, Tokyo 192-0392, Japan

**Keywords:** intranasal brain delivery, glioma, anti-cancer drug, cell penetrating peptides, nanomicelles

## Abstract

The blood-brain barrier is a substantial obstacle for delivering anticancer agents to brain tumors, and new strategies for bypassing it are sorely needed for brain tumor therapy. Intranasal delivery provides a practical, noninvasive method for delivering therapeutic agents to the brain. Intranasal application of nano-sized micelles that have been modified with Tat peptide facilitates brain delivery of fluorescent model materials. In this study, we evaluated a nose-to-brain delivery system for brain tumor therapy. We nasally administered the anti-tumor drug camptothecin (CPT) in solution and in methoxy poly(ethylene glycol) (MPEG)/poly(ε-caprolactone) (PCL) amphiphilic block copolymers (MPEG-PCL) and cell penetrating peptide, Tat analog-modified MPEG-PCL (MPEG-PCL-Tat) MPEG-PCL-Tat to rats bearing intracranial glioma tumors and quantified the cytotoxicity against glioma cells, and the therapeutic effects. CPT-loaded MPEG-PCL-Tat micelles showed higher cytotoxicity than CPT-loaded MPEG-PCL. CPT-free MPEG-PCL-Tat didn’t show any cytotoxicity, even at high concentrations (2 mmol/mL). CPT-loaded MPEG-PCL-Tat micelles significantly prolonged the median survival of rats. These results indicate that intranasal delivery of anti-cancer drugs with cell penetrating peptide-modified nanomicelles might be an effective therapy for brain tumors.

## 1. Introduction

The blood-brain barrier (BBB) is formed by tight junctions within the capillary endothelium of the vertebrate brain [1], and is a substantial obstacle to the delivery of many drugs to the brain. Glioblastomas are the most frequent types of intracranial tumors, being locally infiltrating, aggressive and hypervascularized tumors with a median survival rate of less than one year. The current therapy is cytoreductive surgery followed by radiotherapy, with a more limited role for adjuvant chemotherapy. Glioblastomas are dependent on angiogenesis, as proliferation of microvascular endothelial cells [2,3,4,5]. It is well-known that many chemotherapeutic agents have a low therapeutic index in brain tumors [6]. The failure of chemotherapy is due to the inability of intravenously administered anticancer agents to reach the brain tissue. The BBB is one of the most important obstacles for preventing the penetration of drugs into the central nervous system (CNS) [7]. Chemical modification of drug itself into a more lipophilic and neutral form or create a prodrug is an effective way to delivery of drugs to the brain tumor. The prodrug approach involves the administration of the drug in a form that is inactive or weakly active, but readily able to penetrate the BBB and then to be converted into active form within the brain [6,8]. Consequrntly, there is a great need for new therapeutic strategies that will provide efficient drug delivery to brain tumors.

In the last decade, intranasal administration of drugs has attracted considerable interest as it provides a non-invasive method for bypassing the BBB and delivering therapeutic drugs directly to the central nervous system (CNS) [9,10]. Drugs administered to the nasal cavity can travel along the olfactory and trigeminal nerves to reach many regions within the CNS [1,10,11,12,13]. In addition to bypassing the BBB, intranasal delivery provides rapid delivery of drugs to the CNS, avoids hepatic first-pass drug metabolism, and eliminates the need for systemic delivery, thereby reducing unwanted systemic side effects [1,11]. Intranasal delivery also allows painless and convenient self-administration. Recently, anticancer agents such as methotrexate [14,15], 5-fluorouracil [16], and raltitrexed [17] have been intranasally delivered to the CNS and/or CSF. 

We have previously reported that cell-penetrating peptide-modified MPEG-PCL nanomicelles (MPEG-PCL-Tat) promote cellular uptake into the C6 glioma cells and intranasal brain delivery of fluorescein-model drugs (coumarin). [18]. Therefore, we hypothesized that intranasal delivery of an anti-cancer drug with MPEG-PCL-Tat would enable the compound to reach intracranial tumors and inhibit tumor growth *in vivo* without systemic side effects. In this study, we nasally administered the anti-tumor drug camptothecin (CPT) with MPEG-PCL and MPEG-PCL-Tat to rats bearing intracranial glioma tumors and quantified the cytotoxicity against glioma cells, and the therapeutic effects.

## 2. Results

### 2.1. Characterization

MPEG-PCL block copolymers were synthesized by ring-opening polymerization of ε-CL in the presence of MPEG and characterized by ^1^H-NMR and GPC according to our previous study [18,19]. The molecular weight of the MPEG-PCL block copolymers was 3,718 by GPC (calibrated with PEG standard) and 3,940 from the ^1^H-NMR spectrum. Additionally, the polydispersity index of MPEG-PCL was 1.08. Conjugation of the Tat analog to MPEG-PCL through the ester bond was confirmed using the ninhydrin reaction, which gives a reddish violet coloration upon reaction with the amino residue. The particle size of MPEG-PCL micelles was larger than that of MPEG-PCL-Tat micelles, and the zeta potential of MPEG-PCL micelles was negative, whereas that of MPEG-PCL-Tat was positive (Table 1), suggesting that the Tat analog is present on the surface of the nanoparticles. In addition, as shown in Table 2, the particle size of MPEG-PCL and MPEG-PCL-Tat slightly increased compared with that after CPT-loading and the encapsulation efficiencies of MPEG-PCL and MPEG-PCL-Tat showed 60%.

**Table 1:** Particle diameter and zeta potentialof MPEG-PCL and MPEG-PCL-Tat.

Polymers	Diameter (nm)	Zeta potential (mV)
MPEG-PCL	48.5 ± 3.10	−1.77 ± 0.78
MPEG-PCL-Tat	32.1 ± 5.60	7.26 ± 2.25

mean ± S.D., n = 3.

**Table 2:** Particle diameter, zeta potential and encapsulation efficiency of camptothecin (CPT)-loaded MPEG-PCL and MPEG-PCL-Tat.

Polymers	Diameter(nm)	Zeta potential (mV)	Encapsulation efficiency of CPT (%)
MPEG-PCL	102.8 ± 9.07	−11.7 ± 2.72	56.6 ± 12.6
MPEG-PCL-Tat	88.5 ± 20.2	10.4 ± 2.84	62.5 ± 9.17

mean ± S.D., n = 3.

### 2.2. In Vitro Cytotoxicity

**Figure 1:**
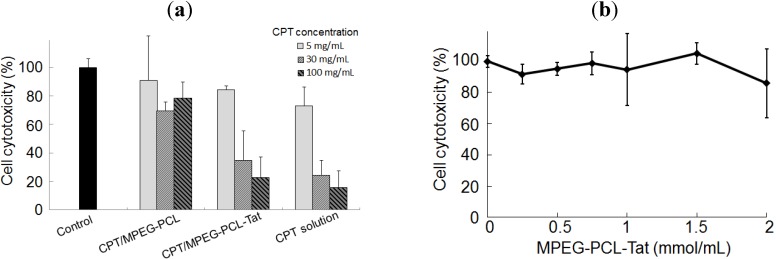
*In vitro* cytotoxicity in C6 cells transfected with camptothecin (CPT). (**a**) Cell cytotoxicity (%) in C6 cells 12 h after transfectionwith untreatment (control; black) and 5 (light gray), 30 (medium gray) and 100 (dark gray hatch) mg/mL of CPT solution, CPT-loaded MPEG-PCL and CPT-loaded MPEG-PCL-Tat. Each bar representsthe mean±S.D. (n=3). (**b**) Cell viability (%) in C6 cells 12 h after transfection with various concentrations of CPT-free MPEG-PCL-Tat. Each bar representsthe mean±S.D. (n=3).

The cells were incubated with different concentrations of CPT solution or CPT-loaded MPEG-PCL and MPEG-PCL-Tat for 12 h and *in vitro* cytotoxicity were analyzed to assess the CPT effect on C6 glioma cells. At each concentration of CPT, cytotoxicity CPT-loaded MPEG-PCL-Tat was higher than with CPT-loaded MPEG-PCL (Figure 1a). Then, CPT-free MPEG-PCL-Tat didn’t show the any toxicity to cells at the high concentration (Figure 1b). These results indicate that Tat-modified micelles have a strong interaction with the C6 cells, and it would therefore be expected that Tat-modified micelles would result in greater delivery of CPT into cells than micelles without Tat.

### 2.3. Therapeutic Effects of Brain Tumor in Glioma Model Rats

**Figure 2:**
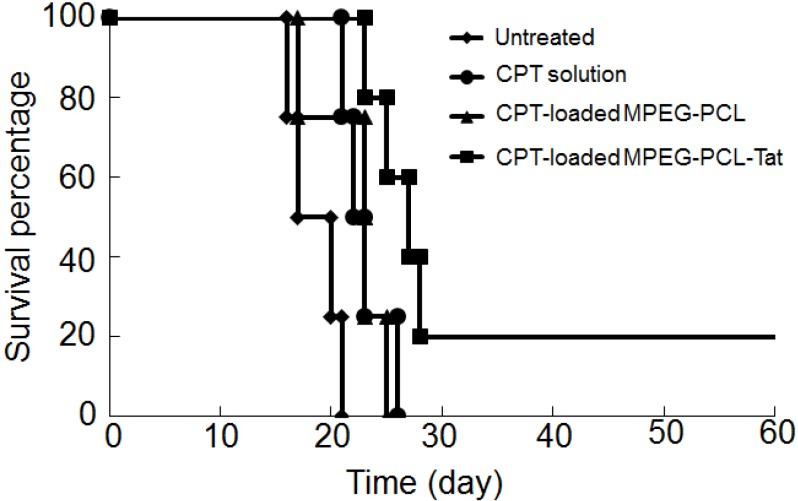
Kaplan-Meier survival curves of intracranial C6 glioma tumor bearing rats after no treatment (diamonds) or treatment with intranasal camptothecin (CPT) solution (circles), CPT-loaded MPEG-PCL (triangles) or CPT MPEG-PCL-Tat (squares). The 1.2 mg/kg as CPT dose were administrated to rats (n=4–5).

The mean survival periods of rats intranasal administrated of various CPT formulations were determined to evaluate the therapeutic effect (Table 3; Figure 2). The mean survival period of the untreated rats was 18.2 days, with all rats dying by 21 days after tumor inoculation The mean survival period of rats treated with CPT solution (23.0 days), CPT-loaded MPEG-PCL (22.0 days), and CPT-loaded MPEG-PCL-Tat (32.6 days) was longer than that of untreated rats. Furthermore, the mean survival period of rats treated with CPT-loaded MPEG-PCL-Tat was longer than that of rats treated with CPT solution and CPT-loaded MPEG-PCL. Then, one of the five rats treated with CPT-loaded MPEG-PCL-Tat survived for more than 120 days after tumor inoculation.

**Table 3:** Median survival period of rats bearing intracranial C6 glioma treated with camptothecin (CPT)-loaded micelles.

Treatment	Number of rats	Number of long-term survivors	Average of survival period (days)	*p* value
Non-treated	4	0	18.2	
CPT solution	4	0	23.0	<0.05 ^a^
CPT-loaded MPEG-PCL	4	0	22.0	<0.05 ^a^
CPT-loaded MPEG-PCL-Tat	5	1 (>120 days)	32.6	<0.01 ^a^, <0.05 ^b,c^

^a^ Comparing non-treated rats, ^b^ Comparing treatment groups with CPT solution, ^c^ Comparing treatment groups with CPT-loaded MPEG-PCL.

**Figure 3:**
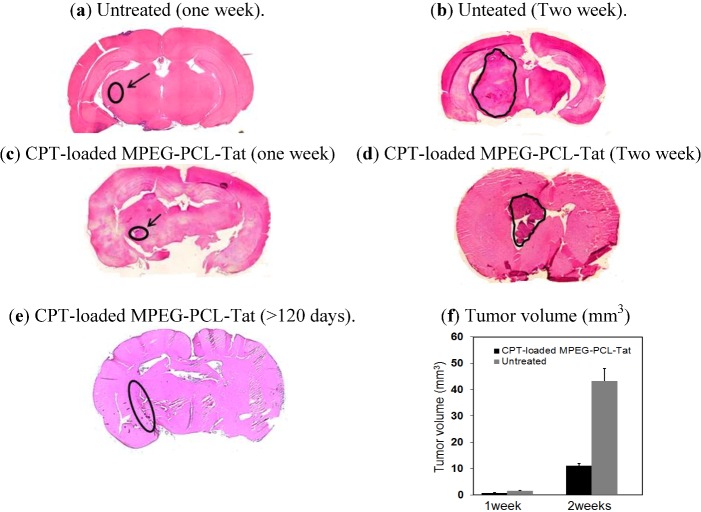
Photographs of HE stained brain tissue from an untreated rat one (**a**) and two (**b**) weeks after and a rat treated with CPT-loaded MPEG-PCL-Tat one (**c**) and two (**d**) weeks and 120 days(**e**) after. (**f**) Tumor volume after one and two weeks of no treatment (grey) or treatment with CPT-loaded MPEG-PCL-Tat (black) (mean±S.E., n=3).

Figure 3a–e shows representative HE stained coronal sections from untreated and treated rats with CPT-loaded MPEG-PCL-Tat micelles. Although the tumor size in rat brain tissue after one week of treatment with CPT-loaded MPEG-PCL-Tat was similar to that in untreated rats, the tumor size after two weeks of treatment with the CPT-loaded MPEG-PCL-Tat was about 4.5 times smaller than in untreated rats (Figure 3f), indicating that CPT-loaded MPEG-PCL-Tat was markedly suppressed tumor growth. 

**Figure 4:**
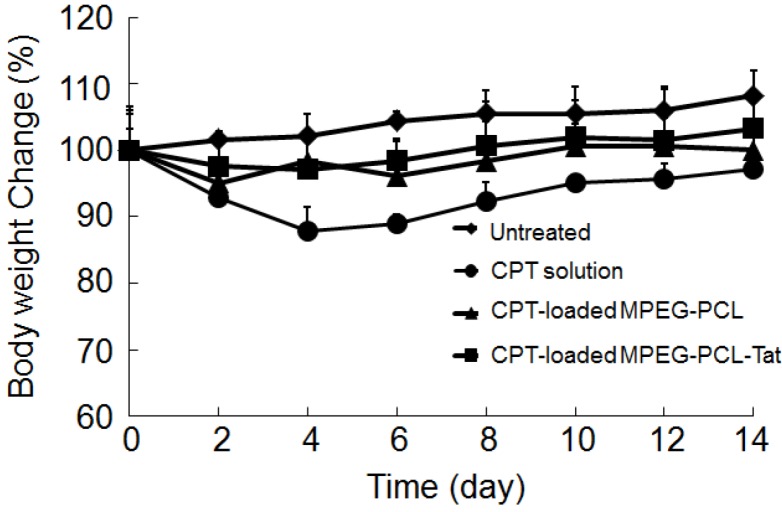
Body weight change in the two weeks after in rats bearing intracranial C6 glioma after no treatment (diamonds) or treatment with intranasal camptothecin (CPT) solution (circles), CPT-loaded MPEG-PCL (triangles) or CPT MPEG-PCL-Tat (squares).

In order to evaluate the systemic side effect of CPT, whole body weight was also monitored (Figure 4). After treatment of CPT solution (CPT was 30 µg/rat), body weight was significantly reduced compared with the untreated rats, indicating that the systemic toxicity of the CPT at the given dose was appeared. In contrast, CPT-loaded MPEG-PCL or CPT-loaded MPEG-PCL-Tat did not cause significant changes in whole body weight, suggesting that the micellar formulations are effective at reducing the systemic drug absorption.

## 3. Discussion

Chemotherapeutic treatment is widely used for brain tumor treatment: however, the outcomes continue to be unsatisfactory. In general, an increase in the local antitumor drug concentration within the tumor may improve the outcome of the drug therapy. Intranasal brain delivery is consistent with the extraneuroral pathway that has been proposed for transport of therapeutic agents from the nasal cavity into the brain. This pathway occurs along the olfactory and trigeminal sensory neurons and likely involves extracellular bulk flow along perineuronal and perivascular routes, delivering the drug directly to the brain parenchyma, spinal cord, and perhaps also the CSF [10]. In addition, in our previous study, we demonstrate that intranasal administration combined with nano-sized micelles as a drug carrier strikingly increased the brain distribution of hydrophobic model compound in glioma bearing rats more than that without nano-sized micelles.

Therefore, we hypothesized that intranasal delivery of an anti-cancer drug with MPEG-PCL-Tat micelles enables the compound to reach intracranial tumors and inhibit tumor growth *in vivo* without systemic side effects. In this study, we nasally administered the anti-tumor drug camptothecin (CPT) with MPEG-PCL and MPEG-PCL-Tat to rats bearing intracranial glioma tumors and quantified the cytotoxicity against glioma cells, and the therapeutic effects.

*In vitro* cytotoxic assay, at each concentration of CPT, cytotoxicity CPT-loaded MPEG-PCL-Tat was higher than with CPT-loaded MPEG-PCL. This may be due to the improvement of the cellular uptake efficiency of CPT-loaded MPEG-PCL-Tat micelles using Tat, which is a cell penetrating peptide, on the surface of micelles. Then, CPT-free MPEG-PCL-Tat didn’t show the any toxicity to cells at the high concentration. These results indicate that Tat-modified micelles have a strong interaction with the C6 cells, and it would therefore be expected that Tat-modified micelles would result in greater delivery of CPT into cells than micelles without Tat.

*In vivo* therapeutic studies, Tat-modified MPEG-PCL micelles achieved strongly therapeutic efficiency after seven days of continuous delivery. In our previous report, MPEG-PCL-Tat could promote the intracellular and nose-to-brain delivery of hydrophobic fluorescein-model drugs [19]. Therefore, in this study, MPEG-PCL-Tat also promoted the delivery of CPT into the brain and the mean survival time clearly increased. These results suggest that a Tat-modified nanoparticle can be due to high penetration of CPT at the nasal epithelia, and the specificity achieved with intranasal delivery appears to be superior to the results obtained using simple CPT solution, which reportedly delivers drugs in the blood circulation.

## 4. Experimental Section

### 4.1. Materials, Cells and Rats

We purchased poly(ethylene glycol) (MPEG, Mn = 2,000 Da) from Sigma-Aldrich Co., MO, USA. ε-Caprolactone was purchased from Tokyo Kasei (Tokyo, Japan) and anti-tumor drug, camptotecin (CPT), was from Wako Pure Chemical Industries, Ltd. (Osaka, Japan). Tat-glycine peptide (Tat-G) (10-mer: GlyArgLysLysArgArgGlnArgArgArg) was synthesized using the Fmoc strategy according to our previous study [18,19]. The copolymers of MPEG-polycaprolactone (PCL) (MPEG-PCL) were synthesized by the ring opening polymerization of ε-caprolactone initiated by MPEG (Mn 2,000 Da). Synthesis of Tat-conjugated MPEG-PCL (MPEG-PCL-Tat) through the ester bond was performed as described in our previous papers [18,19]. Briefly, Tat-G (0.02 mmol) and MPEG-PCL (0.02 mmol) were dissolved in dichloromethane, and then WSCI (0.02 mmol) and 4-dimethylaminopyridine (0.02 mmol) were added and reacted. After 24 h, the reaction solution was evaporated and dialyzed in distilled water using a dialysis tube (3,500 MW, Spectrum Laboratories, Inc., Rancho Dominguez, CA USA) for 24 h to remove non-reacted Tat-G. After freeze-drying, MPEG-PCL-Tat was obtained.

C6 rat glioma (C6) cells were purchased from ATCC (Baltimore, MD, USA). Cell culture medium, F-12K nutrient mixture (F-12K), certified fetal bovine serum (FBS), penicillin/streptomycin stock solutions, and 0.25% Trypsin-EDTA were purchased from Life technologies Japan Co. (Tokyo, Japan).

Seven-week-old Sprague-Dawley (SD) male rats were purchased from Japan SLC Inc. (Shizuoka, Japan). The rats were housed under standard conditions of temperature (22–24 °C), humidity (40%–60%), and 12 h light/dark-cycles with the light period starting at 08:00. Food and water were supplied ad libitum. All experiments with animals were carried out in accordance with a protocol approved by the Animal Care and Ethics Committee of Tokyo University of Pharmacy and Life Sciences (TUPLS).

### 4.2. Cell Culture

Rat C6 glioma cells were maintained in F-12 K supplemented with 10% FBS, 1% penicillin/streptomycin at 37 °C, 5% CO_2_. The F-12K was. Cells were seeded into culture flasks for seven days. The cells were harvested by trypsinization, washed by PBS, and resuspended in F-12K medium. 

### 4.3. Preparation of CPT-Loaded Micelles

MPEG-PCL and MPEG-PCL-Tat micelles were prepared by self-assembly. Camptothecin (CPT) was loaded into the micelles using a water-in-oil (w/o) emulsion method. Briefly, 1 mL of w/o emulsion (180 mg/mL of MPEG-PCL or MPEG-PCL-Tat in 5 mL of dichloromethane/chloroform/ methanol (5/4/1) containing 1.8 mg/mL of CPT) was sonicated for three minutes, and the solvent was evaporated. Then, appropriate quantities of distillated water were added, and the suspension was filtered by syringe filter (pore size 0.2 μm; Advantec MSF, Inc.) to remove the unentrapped CPT. Finally, the CPT-loaded micelles were freeze-dried and stocked at −20 °C. The CPT-loaded micelles were dissolved in F-12K medium (*in vitro* assay) or ultrapure-water (*in vivo* assay) at the time of experiments. The level of CPT entrapped in the micelles was analyzed by high-performance liquid chromatography (HPLC, Class LC-10; Shimadzu, Kyoto, Japan) with the detector (SPD-10A), pump (LC-10AT), auto-injector (SIL-10_XL_), column oven (DGU-12A) and module (CBM-10A) at a flow rate of 1.0 mL/min and column temperature of 40 °C and methanol/acetonitrile/50 mM KH_2_PO_4 _solution (35/15/50) were used as the mobile phase. The percent CPT encapsulation was determined as follows: % encapsulation = 100 × (measured CPT amount/theoretical CPT amount) [20].

### 4.4. Physiochemical Characterization

The mean particle size and zeta potential of the various micelles were determined by dynamic light scattering (DLS)-700 unit (Otsuka Electronics Co., Ltd., Osaka, Japan) and a Zeta Potential/Particle Sizer NICOMPTM 380 ZLS (Nicomp Particle Size Systems, Santa Barbara, CA, USA).

### 4.5. Cytotoxicity in C6 Glioma Cells Transfected with Various CPT Preparations

Rat C6 glioma cells were seeded in a 96-well plate at a density of 20,000 cells per well and were allowed to adhere to the plate for 24 h. The cells were washed two times by PBS and treated with CPT solution dissolved in FBS-free F-12K containing 30% DMSO, CPT-loaded MPEG-PCL or MPEG-PCL-Tat in FBS-free F-12K at various CPT concentrations for 12 h at 37 °C and 5% CO_2_. After 12 h, the cells were incubated with CCK-8 solution for 3 h. The absorbance of viable cells was measured at 450 nm using a microplate reader. The absorbance of control cells indicated 100% cell viability.

### 4.6. Intracranial Model of C6 Glioma

For intracranial tumor establishment, C6 glioma cells were harvested by trypsinization, washed two times in PBS and resuspended in F-12K medium. Rats were anesthetized by intra-peritoneal injection of pentobarbital (50 mg/kg). The dorsal surface of the head was disinfected with iodine and wiped with 70% alcohol. The skin was incised at midline and retracted. The area around the site of injection was blotted dry. A small hole was drilled 3 mm anterior to the lambda and 3 mm lateral to the midline. Each rat was injected slowly (over approximately three minutes) with 1 × 10^6^ C6 glioma cells in 10 μL F-12K medium, through a syringe fitted with a 30-gauge needle inserted to a depth of 7 mm in the left cerebral hemisphere. Finally, the divided ends of skin were sutured back together. The animals were monitored for the hour after the surgery and then daily. 

### 4.7. Therapeutic Efficacy Studies

The tumor-bearing rats were randomly divided into four groups of four or five rats each. One group received no treatment. The other groups received treatment with CPT suspension, CPT-loaded MPEG-PCL, or CPT-loaded MPEG-PCL-Tat at the dose of 1.2 mg/kg as CPT amounts. Treatment was administered with intranasal injections once a day for one week. The survival time of each rat was recorded. The survival was evaluated using Kaplan-Meier method. The mean survival period was defined as the day at which survival was 50%. 

In addition, in order to estimate tumor growth, the sections of brain tissue were prepared. Actually, rats after no treatment or treatment with CPT-loaded MPEG-PCL-Tat were killed and the brain tissues were removed and fixed in parafin. The paraffin-embedded brains were sliced into serial coronal sections (15 μm thick) using a sliding microtome (TYPE NS-31 Yamato Koki Co., ltd., Miyazaki, Japan), and stained with hematoxylin and eosin (HE). The coronal section with the maximum timorous area was photographed with a digital microscope (BZ-8100, Keyence Corporation, Tokyo, Japan) for quantifying tumor growth. The HE stained coronal sections were observed by microscope to determine the tumor size. Tumor The size of tumor tissue was measured by vernier calipers and tumor volume was calculated using the formula below, where length is the longer axis of the tumor: 


Tumor volume = (length × (width)^2^)/2

Furthermore, in order to evaluate the systemic side effects of CPT, the body weight was also recorded prior to the surgery and at regular intervals after surgery. Food and water were supplied ad libitum.

### 4.8. Statistical Analysis

Data from the *in vitro* experiments are expressed as mean ± standard deviation (S.D.). Data from the *in vivo* experiments are expressed as mean ± standard error (S.E.). Statistical analysis was performed using an unpaired Student’s t-test for two groups. Statistical analysis of the survival period was performed using a non-parametric log-rank Test.

## 5. Conclusions

This study has demonstrated the new finding that a combination of intranasal administration and Tat-modified MPEG-PCL achieved a striking inhibition of tumor growth, and prolonged survival of C6 glioma bearing rats without any toxicity. However, intranasal delivery of Tat-modified MPEG-PCL might deliver drugs beyond the tumor boundary into adjacent normal brain tissues. Therefore, it is necessary to develop an intranasal delivery system that prevents unwanted damage to normal brain tissues adjacent to the brain tumor.
